# Nutrition support therapy prescribing practices in hospice and palliative care units: a retrospective cohort study investigating physician prescribing practices and roles of pharmacists at a tertiary cancer center

**DOI:** 10.1186/s43046-025-00297-9

**Published:** 2025-07-07

**Authors:** Nadine N. Abdelhadi, Saad Jaddoua

**Affiliations:** 1 Department of Clinical Pharmacy and Pharmacy Practice, Faculty of Pharmacy, Aqaba University of Technology, Aqaba, Jordan; 2 King Hussein Cancer Center, Amman, Jordan

**Keywords:** Nutrition support therapy, Cancer, Clinical pharmacy, Pharmacy practice, Cancer

## Abstract

**Background:**

The literature on nutrition support therapy prescribing practices by physicians and the roles of nutrition support pharmacists in palliative and hospice care cancer patients is limited.

**Methods:**

The study aimed to analyze the prescribing practices of physicians and the roles of clinical pharmacists at a tertiary cancer center. A retrospective analysis of 12527 electronic records of hospice and palliative care cancer patients. All nutrition support therapy prescriptions by physicians and clinical pharmacists’ interventions were recorded. Analysis was conducted utilizing the Jamovi statistical package 2022.

**Results:**

The study population comprised inpatients and homecare patients. The most frequently prescribed nutrition support therapy was vitamins and minerals supplements, followed by enteral nutrition and parenteral nutrition. The total number of nutrition support pharmacist interventions was 660 (5.2%). The acceptance rate of interventions by physicians was 90%. Initiating mineral use was the most frequent intervention, followed by discontinuation of mineral use.

**Conclusion:**

Vitamins and mineral supplements are the most prescribed type of nutrition support therapy. The interventions of clinical pharmacists were highly accepted by physicians. Initiating mineral use is the most frequent intervention. Further research is needed to explore the impact of nutrition support therapy on patient outcomes and barriers to its implementation.

## Background

Palliative care is specialized care indicated for patients living with a serious chronic illness, such as cancer, chronic obstructive pulmonary disease (COPD), or heart failure [1, 2]. Palliative care's main goal is to improve patients'quality of life, and it is best provided soon after the diagnosis of disease, but it is helpful at any stage of illness [3]. On the other hand, hospice care focuses on the quality of life of a patient with a serious terminal illness who is not responding to treatment [4]. Both palliative and hospice care provide comprehensive services to support patients and improve their quality of life, but in hospice, attempts to cure the patients are stopped [4]. Guidelines recommend assessing nutritional deficiencies in all cancer patients, as malnutrition leads to poor prognosis and decreased patient quality of life [5]. Cancer and its treatment are often associated with tumor-induced anorexia, diminished nutritional intake, and weight loss [6, 7]. The role of nutritional support for cancer patients in palliative care is still controversial [8–11].

Nutrition support therapy is a component of medical nutrition therapy that can include oral, enteral, and parenteral nutrition to treat or prevent malnutrition [12]. Enteral nutrition (EN) is a type of nutrition support therapy in which liquid formulations are administered directly into the stomach or small intestine when patients are unable to attain an adequate oral intake [12, 13]. Parenteral nutrition (PN) is another type of nutrition support therapy in which nutrients are administered intravenously [14]. PN is indicated when the gastrointestinal tract is inaccessible or when the nutritional needs of patients cannot be met through the gastrointestinal tract alone [14].

Micronutrients are nutrients needed by the body in minute amounts, such as vitamins and minerals [15]. Deficiency or excess of micronutrients can lead to hemodynamic imbalance, abnormalities, and even life-threatening conditions [15]. Few studies explored the role of clinical pharmacists in managing micronutrient deficiencies in hospital settings [16–20].

Nutritional support improves the quality of life of early-stage palliative cancer patients, minimizes the impact of catabolism associated with cancer, prolongs survival, and decreases health costs [8–11]. It should be considered early for malnourished cancer patients [5]. A multidisciplinary team approach is utilized in the nutritional management of end-stage cancer patients [9]. The multidisciplinary team is usually composed of physicians, pharmacists, nutritionists, and other healthcare professionals [13]. Clinical Nutrition support pharmacists (NSP) are healthcare professionals specializing in managing nutrition support therapy (NST) [21]. They provide various services, including evaluation of nutritional status, compounding, and monitoring parenteral nutrition and enteral nutrition [13]. Clinical pharmacists are an integral part of the cancer care [16]. Comprehensive pharmaceutical care cost-effectively improved cancer patients’ outcomes, and the roles of clinical pharmacists continue to expand [16–20].

This study aimed to explore nutrition support therapy prescribing practices by physicians and the roles of clinical pharmacists in advanced cancer patients admitted to hospice and palliative care units.

## Methods

### Study design, participants, and study procedures

A retrospective analysis of 12527 electronic records of hospice and palliative care cancer patients at King Hussein Cancer Center (KHCC) from January 2020 to December 2022 was conducted. All nutrition support therapy prescriptions by physicians and clinical pharmacists’ interventions were recorded. The number of included records was 660 (5.3%). The study follows the EQUATOR Network guideline -STROBE checklist.

This study was approved by the institutional review board (IRB) at KHCC on October 25, 2021, with the approval number RC/2021/153.

### Statistical analysis

Descriptive statistics were used to evaluate the findings in frequencies and percentages.

The Mann–Whitney U test was used to compare the means of the time nutrition support clinical pharmacists took to intervene between the hospice and palliative care units and between the inpatient and home care. Jamovi statistical package 2022 was used to conduct the analysis [22, 23]. A *p*-value less than 0.05 was considered statistically significant.

## Results

The total number of nutrition support pharmacy interventions was 660 (5.2%). The median age of subjects was 61.0 years (interquartile range: 50–71). The study population comprised female patients (*n* = 305, 46.2%) and adult patients (*n* = 355, 53.8%) (Table 1).

**Table 1:** Participants’ median age according to gender

Percentiles							
	Gender	N	Median	IQR	25 th	50 th	75 th
Age	F	305	64	19.0	55.0	64.0	74.0
	M	355	57	20.0	46.0	57.0	66.0

### Distribution of participants based on service

The majority of participants were admitted in 2022 (*n* = 311, 47.1%). The number of patients admitted in 2020 was (*n* = 135, 20.5%), and in 2021 was (*n* = 214, 32.4%).

### Distribution of participants based on type of service

Most patients were admitted to palliative care units (*n* = 552, 83.6%), followed by hospice care services (*n* = 108, 16.4%). The study population comprised inpatients (*n* = 629, 95.3%) and homecare patients (*n* = 31, 4.7%).

### Analysis of nutrition support therapies prescribing in hospice and palliative care patients

The total number of patients’ records with nutrition support therapy prescriptions was 660. The most frequently prescribed nutrition support therapy was vitamins and minerals supplements (*n* = 540, 81.8%), followed by EN (*n* = 92, 13.9%) and PN (*n* = 28, 4.2%).

### Analysis of vitamin prescribing by physicians

Total number of patients’ records with vitamin use was 195. Multivitamin (*n* = 87, 44.6%) was the most frequently prescribed by physicians, followed by Vitamin B (*n* = 46, 23.6%). The frequencies of vitamin prescribing are described in Table 2.

**Table 2:** Frequencies of vitamin prescriptions by physicians

Vitamin	Counts	% of Total	Cumulative %
Multivitamin/Mineral Supplement	87	44.6%	44.6%
Vitamin B	46	23.6%	68.2%
Vitamin C	15	7.7%	75.9%
Vitamin D	40	20.5%	96.4%
Vitamin E	7	3.6%	100.0%

### Analysis of vitamin prescribing by physicians

The frequencies of vitamin prescribing are described in Table 3.

**Table 3:** Frequencies of vitamin prescription by physicians

Mineral	Counts	% of Total	Cumulative %
Calcium Carbonate	35	8.1%	8.1%
Calcium Gluconate	17	3.9%	12.0%
Calcium Polystyrene Sulfonate	15	3.5%	15.5%
Magnesium Chloride	1	0.2%	15.7%
Magnesium Oxide	9	2.1%	17.8%
Magnesium Sulfate	36	8.3%	26.2%
Multivitamin/Mineral Supplement	87	20.1%	46.3%
Potassium Chloride	118	27.3%	73.6%
Potassium Glucoheptonate	6	1.4%	75.0%
Potassium Gluconate	3	0.7%	75.7%
Potassium Phosphate	13	3.0%	78.7%
Sodium Acetate	1	0.2%	78.9%
Sodium Alginate	4	0.9%	79.9%
Sodium Bicarbonate	12	2.8%	82.6%
Sodium Chloride	27	6.3%	88.9%
Sodium Hyaluronate	3	0.7%	89.6%
Sodium Phosphates	41	9.5%	99.1%
Zinc Oxide	4	0.9%	100.0%

The total number of patient records with the use of EN and PN was 120. Enteral nutrition (*n* = 92, 76.7%) was found to be the most used class of nutrition support therapies, followed by total parenteral nutrition (*n* = 28, 23.3%).

#### Analysis of clinical nutrition support pharmacists’ interventions

The total number of nutrition support pharmacy interventions was 660 (5.2%). The acceptance rate of clinical nutrition support pharmacists’ interventions by physicians was 90%. Initiating mineral use (*n* = 139, 21.1%) was the most frequent intervention, followed by discontinuation of mineral use (*n* = 97,14.7%). Table 4 depicts the frequencies of interventions.

**Table 4:** Frequencies of intervention by service type

Intervention	Service type	Counts	% of Total	Cumulative %
Allergy prevented	Homecare	0	0.0%	0.0%
	Inpatient	1	0.2%	0.2%
Clarification of orders	Homecare	0	0.0%	0.2%
	Inpatient	12	1.8%	2.0%
Conversion from Intravenous (IV)-to-PO route done	Homecare	0	0.0%	2.0%
	Inpatient	1	0.2%	2.1%
Conversion from Intravenous (IV)-to-PO route recommended	Homecare	0	0.0%	2.1%
	Inpatient	1	0.2%	2.3%
Discontinuation of enteral nutrition	Homecare	0	0.0%	2.3%
	Inpatient	61	9.2%	11.5%
Discontinuation of mineral use	Homecare	0	0.0%	11.5%
	Inpatient	97	14.7%	26.2%
Discontinuation of multivitamin	Homecare	4	0.6%	26.8%
	Inpatient	71	10.8%	37.6%
Discontinuation of total parenteral nutrition	Homecare	0	0.0%	37.6%
	Inpatient	3	0.5%	38.0%
Discontinuation of vitamin use	Homecare	22	3.3%	41.4%
	Inpatient	55	8.3%	49.7%
Dose clarified/evaluated	Homecare	0	0.0%	49.7%
	Inpatient	66	10.0%	59.7%
Duration of Order Clarified	Homecare	0	0.0%	59.7%
	Inpatient	10	1.5%	61.2%
Infusion Rate Clarified	Homecare	0	0.0%	61.2%
	Inpatient	11	1.7%	62.9%
Initiation of Enteral nutrition	Homecare	0	0.0%	62.9%
	Inpatient	3	0.5%	63.3%
Initiation of Mineral use	Homecare	0	0.0%	63.3%
	Inpatient	139	21.1%	84.4%
Initiation of Multivitamin use	Homecare	0	0.0%	84.4%
	Inpatient	1	0.2%	84.5%
Initiation of Vitamin use	Homecare	0	0.0%	84.5%
	Inpatient	3	0.5%	85.0%
Initiation of total parenteral nutrition	Homecare	0	0.0%	85.0%
	Inpatient	9	1.4%	86.4%
Lab Evaluation	Homecare	0	0.0%	86.4%
	Inpatient	25	3.8%	90.2%
Medication Reconciled/Discharge	Homecare	2	0.3%	90.5%
	Inpatient	16	2.4%	92.9%
Medication Reconciled/Transfer	Homecare	0	0.0%	92.9%
	Inpatient	13	2.0%	94.8%
Medications reconciled/admission	Homecare	1	0.2%	95.0%
	Inpatient	19	2.9%	97.9%
Renal dose evaluation	Homecare	2	0.3%	98.2%
	Inpatient	1	0.2%	98.3%
The route of administration was clarified	Homecare	0	0.0%	98.3%
	Inpatient	3	0.5%	98.8%
TPN evaluation/adjustment/monitoring	Homecare	0	0.0%	98.8%
	Inpatient	1	0.2%	98.9%
Therapeutic duplication avoided	Homecare	0	0.0%	98.9%
	Inpatient	5	0.8%	99.7%
Therapeutic interchange done	Homecare	0	0.0%	99.7%
	Inpatient	2	0.3%	100.0%

### Analysis of time taken by clinical pharmacists to intervene

The total number of clinical pharmacists'interventions in the management of hospice and palliative care patients was 12527 interventions. Clinical pharmacists performed 660 (5.3%) interventions related to nutrition support therapies. The sum of the times taken was 11,904 min. The median time was 15.0 min (interquartile range: 15–25). The minimum time was 1, and the maximum time was 65 min.

### Time taken by clinical pharmacists to intervene in managing nutrition support therapies in hospice care patients compared to and palliative care patients

The means of time clinical pharmacists took to intervene in the hospice care patients group was compared to the palliative care patients group using the Mann–Whitney U T-test. The test showed a significant difference between the population means (*p*-value < 0.001).

## Discussion

Nutritional interventions are becoming widely used in oncology. However, the purposes and outcomes of such interventions in cancer patients admitted to palliative and hospice care departments are not always well-defined since nutrition is traditionally considered a palliative treatment to be confined to the area of palliative care [24]. A recent study indicated that early supplemental nutrition support improves compliance of patients with oncological disorders [25]. Palliative cancer care patients may live for a long time, but malnutrition worsens the prognosis [24]. In the current research, multivitamins were the most frequently prescribed by physicians, followed by Vitamin B. Recent studies reported that multivitamin use was not associated with survival outcomes and it showed that the associations between survival outcomes and supplementation of vitamins during chemotherapy are consistent with recommendations for caution among cancer patients during chemotherapy, when considering the use of supplements, other than a multivitamin [26, 27]. The current evidence on the efficacy of most dietary factors, including vitamins, appears inadequate to recommend their use [28]. The European Society for Clinical Nutrition and Metabolism (ESPEN) recommends that all cancer patients should be screened for the risk malnutrition. However, nutritional treatments should be used cautiously in patients with advanced cancer disease. Nutritional therapy should be accompanied by exercise training [5]. The impact of vitamin and mineral use on the quality of life of hospice and palliative care patients should be further explored.

A recent research reported the clinical benefits of EN and PN for patients with advanced cancer [25]. However, the current study indicated that the least prescribed nutrition support therapies by physicians were EN and PN. Parenteral and enteral nutrition therapies are uncommon practices in Jordan and can only be reported at King Hussein Cancer Center, a comprehensive cancer center. A recent study indicated that PN is suitable for restoring calorie deficit without an increased symptom burden for palliative [29].

The barriers to the application of these practices would include the shortage of well-trained specialists in enteral and parenteral nutrition. In addition, The study plans for undergraduate medical and pharmaceutical programs in Jordan don’t include a sufficient number of courses in the field of nutrition support therapy. The top management support to adopt these services at Jordanian hospitals, which is necessary for the successful implementation of nutrition support pharmacy services, is currently insufficient. The barrier to implementing these specialized services to cancer patients should be further explored.

This study investigated the role of clinical pharmacists in managing nutrition support therapies in hospice and palliative care cancer patients. The total number of clinical pharmacists'interventions in the management of hospice and palliative care patients was 12527 interventions. The total number of patients’ records with nutrition support therapies was 660. The number of interventions related to nutrition support therapies was 660 (5.3%), which is relatively low when compared to the total number of interventions. Previous studies reported that pharmacists'nutrition knowledge and practice were variable [30–34]. A recent study also reported that most pharmacists are willing to participate in further nutrition education to support their patients [33]. The findings in the literature suggest that barriers to providing nutrition care in the pharmacy setting could include time constraints and renumeration [32–34].

Most patients were admitted to palliative care units followed by hospice care services. There was a significant difference between the time taken by clinical pharmacists to intervene in hospice care patients when compared to palliative care patients. This could be explained by the differences between these patient groups. None of the home care patients were prescribed EN or PN.

Recent studies investigated combined nutrition and exercise program comprising home based palliative care services. It included at-home physical activity prescription, behaviour change education, and nutrition and palliative care consultations. Patients reported improved symptom burden, fatigue, physical activity, dietary intake, and physical function were assessed. Post-intervention interviews examined participant perspectives. The research findings indicated that the multimodal intervention improved protein intake and to the general well-being of the patient by ameliorating vomiting and nausea. This underscores the importance of home-based nutritional treatments in patients admitted to palliative care units [34].

In the current study, none of the home care patients were prescribed EN or PN. More research is needed to explore the barrier to providing specialized nutrition therapies such as enteral and parenteral nutrition in home care settings.

In the present study, the acceptance rate of clinical pharmacists’ interventions by physicians in the current study was high when compared to the acceptance rate reported in recent research on daily hospital practice [33]. This reflects physicians'high level of confidence in clinical pharmacists at KHCC. The role of clinical pharmacists and the impact of nutrition support pharmacy services on the clinical outcomes of patients and healthcare costs should be further studied.

To our knowledge, this is the first study to investigate the role of clinical pharmacists in the management of nutrition support therapy in advanced-care cancer patients admitted to hospice and palliative care units. It is a strength of the present study. More studies are needed to investigate the impact of nutrition support pharmacy practices on advanced cancer patients'quality of life. The cost-effectiveness of these practices could be explored as well.

This is also the first study to explore the mean time the clinical pharmacists took to intervene in hospice and palliative care cancer patients. This is another strength of the study. The clinical pharmacists took between 1 and 65 min to intervene in the management of nutrition support therapies in the present study compared to 15–30 min in another study conducted at a university hospital [20]. The variables affecting the time taken by clinical pharmacists to provide nutrition support pharmacy interventions or other clinical pharmacy services should be studied in future research.

The potential roles of the specialized nutrition support pharmacy practices in improving the quality of life of cancer patients admitted to hospice and palliative care units should be explored in further studies.

The limitations of the present study, which should be acknowledged is the recall bias and the reporting inaccuracies inherent to the retrospective design. Additionally, retrospective designs are more susceptible to confounding factors, which may not have been measured or controlled for at the time of data collection.

### Future research perspectives

Multidisciplinary Teams including physicians, nurses, pharmacists, dietitians, psychologists, social workers, and other healthcare professionals are essential in delivering holistic care [35, 36]. Future studies should evaluate collaborative models that integrate nutrition more explicitly into care planning.

Artificial intelligence (AI) has transformative potential in personalizing nutritional care and predicting outcomes. It can predict nutritional deterioration, cachexia risk, or treatment-related side effects using clinical data [37]. In addition, the Internet of Things (IoT) can provide continuous monitoring of nutritional parameters using smart sensors to track weight, nutritional status and dietary intake. Remote patient monitoring (RPM) tools for early detection of nutritional decline enable timely interventions in high-risk patients [38]. Future research should focus on creating a patient-centered framework that integrate expertise with digital intelligence to enhance patient outcomes.

## Conclusion

PN and EN are uncommonly prescribed in hospice and palliative care cancer patients in Jordan. Vitamins and mineral supplements were the most prescribed nutrition support therapies by physicians. The interventions of clinical pharmacists were highly accepted by physicians. Initiating mineral use is the most frequent intervention, followed by discontinuation of mineral use. The recommendations of clinical pharmacists in the management of nutrition support therapy were well accepted by physicians. Initiation of Mineral use was the most frequent intervention, followed by the discontinuation of mineral use. The least frequently prescribed nutrition support therapy was EN and PN.

Further research is needed to investigate the barriers to implementing nutrition support pharmacy services in hospice and palliative care settings and to explore the effects of these services on patient outcomes.

## Data Availability

No datasets were generated or analysed during the current study.
